# Single-Molecule Imaging and Computational Microscopy Approaches Clarify the Mechanism of the Dimerization and Membrane Interactions of Green Fluorescent Protein

**DOI:** 10.3390/ijms20061410

**Published:** 2019-03-20

**Authors:** Xiaohua Wang, Kai Song, Yang Li, Ling Tang, Xin Deng

**Affiliations:** 1 Key Laboratory of Plant Resources, Institute of Botany, Chinese Academy of Sciences, Beijing 100093, China; wangxh@ibcas.ac.cn (X.W.); liyang184@mails.ucas.edu.cn (Y.L.); tangling181@mails.ucas.edu.cn (L.T.); 2 Chang’an University, Middle Sect South Er Huan Rd, Xi’an 710064, China; songkai@chd.edu.cn; 3 University of Chinese Academy of Sciences, Beijing 100049, China

**Keywords:** single molecule, stoichiometry, molecular dynamics, *N*-myristoylation

## Abstract

Green fluorescent protein (GFP) is widely used as a biomarker in living systems; however, GFP and its variants are prone to forming low-affinity dimers under physiological conditions. This undesirable tendency is exacerbated when fluorescent proteins (FP) are confined to membranes, fused to naturally-oligomeric proteins, or expressed at high levels in cells. Oligomerization of FPs introduces artifacts into the measurement of subunit stoichiometry, as well as interactions between proteins fused to FPs. Introduction of a single mutation, A206K, has been shown to disrupt hydrophobic interactions in the region responsible for GFP dimerization, thereby contributing to its monomerization. Nevertheless, a detailed understanding of how this single amino acid-dependent inhibition of dimerization in GFP occurs at the atomic level is still lacking. Single-molecule experiments combined with computational microscopy (atomistic molecular dynamics) revealed that the amino group of A206 contributes to GFP dimer formation via a multivalent electrostatic interaction. We further showed that myristoyl modification is an efficient mechanism to promote membrane attachment of GFP. Molecular dynamics-based site-directed mutagenesis has been used to identify the key functional residues in FPs. The data presented here have been utilized as a monomeric control in downstream single-molecule studies, facilitating more accurate stoichiometry quantification of functional protein complexes in living cells.

## 1. Introduction

The use of green fluorescent protein (GFP), originally isolated from the *Aequorea victoria* jellyfish, and its derivatives has tremendously increased our knowledge of biological processes with an unprecedented level of detail in living cells [1,2,3]. Expressed as an in-frame fusion to a protein of interest, GFP allows visualization of the molecular behavior and intracellular trafficking of that protein within a living system. Due to the importance of GFP, the molecular structure of *Aequorea* GFP has been characterized extensively. Notably, all GFP-like proteins and their derivatives have a tendency to oligomerize at high concentrations under certain physiological conditions [2,4]. In the orange, red, and far-red parts of the spectrum (emission peaks beyond 550 nm), all naturally-available fluorescent proteins (FPs) are dimeric or tetrameric, even at very low concentrations [3,5]. This property may cause mistargeting and aggregation of fused constructs, rendering these FPs generally unsuitable as fusion tags for studying the localization, interaction, and motility of proteins of interest. It is also important to note that application of FPs for labeling the plasma membrane, whole cells, and tissues, as well as visualization of large organelles (i.e., vacuole and nucleus), does not necessarily require monomeric FPs.

Numerous studies have shown that the weak interaction between *Aequorea* FPs is not sufficient to drive dimerization within the cell in the absence of fusion to other directly-interacting or tightly-clustered proteins. However, some GFP derivatives, such as cyan (CFP) and yellow (YFP) FPs, have a tendency to cause an artifactual fluorescence resonance energy transfer (FRET) reaction on membranes due to their weak dimerization ability [6]. Moreover, if the protein of interest is an oligomer itself, fusion constructs harboring a dimeric or tetrameric FP may result in a network of interacting proteins leading to aggregation [7,8]. Therefore, for the labeling of most proteins, an FP must be monomeric; otherwise, oligomerization of a chimeric construct would interfere with the normal function and localization of the protein of interest. Most importantly, when carrying out single-molecule imaging experiments and step-wise photobleaching-based determination of subunit stoichiometry, fusion constructs with a dimeric or oligomeric FP may form large aggregations, resulting in overestimation of the molecular brightness, cluster size, and subunit counts [9,10].

In wild-type GFP, the dimer interface includes hydrophobic residues Ala206, Leu221, and Phe223, as well as hydrophilic contacts involving Tyr39, Glu142, Asn144, Ser147, Asn149, Tyr151, Arg168, Asn170, Glu172, Tyr200, Ser202, Gln204, and Ser208 [1]. By mutating the neutral alanine residue at position 206 to a positively-charged lysine residue (A206K), Zacharias et al. efficiently minimized the interaction between two GFP molecules [2]. Although most FPs exist as very weak dimers, they can be made truly monomeric simply by introducing this A206K point mutation, generally without deleterious effects [8,11]. This mutation disrupts the dimerization interface, reducing the dimerization binding affinity by 740-fold to 74 mM [2]. Despite the importance of this interaction, the local conformation of the GFP dimerization interface remains poorly understood. It is therefore crucial to understand the nature of the structural changes affecting binding between certain amino acids at the atomic level.

The crystal structure of GFP provides important information regarding the overall dimerization, atomistic interactions, and contacts between monomers. However, many of the details regarding how conformational flexibility and structural changes affect the key interactions responsible for the formation of dimers remain elusive despite extensive studies. Molecular dynamics (MD) is one of the most appropriate and broadly-implemented methods for studying dynamic changes in protein structure and interactions, providing atomistic insights that cannot be obtained experimentally [12,13,14,15]. MD simulations may serve as a computational microscope, revealing important biomolecular mechanisms at spatial and temporal scales that are difficult to observe experimentally. Several studies have explored the internal flexibility and properties of the chromophore inside GFP using MD simulations; however, most of these studies have employed coarse models and methods that do not take into account the atomistic details [16,17], whereas those should use atomic-level descriptions. As there remains no available experimentally-derived structure of this key A206K mutation in GFP, atomistic MD may be a fast and reliable alternative method to provide this vital missing structural information in dimeric formation.

In this study, we employed a combination of experimental and fully-atomistic MD simulation approaches to assess the conformational dynamics and noncovalent dimerization equilibrium on a nanosecond timescale, along with single molecular characterization of the reversible protein–protein aggregation, binding, and subunit counting. Our single-molecule-level results showed that the A206K mutation greatly reduced the rate of GFP dimerization. Furthermore, MD simulations were carried out to monitor the conformational dynamics induced by the A206K mutation compared with the wild-type GFP and the membrane insertion of *N*-myristoylated-GFP. The results of atomic MD simulations indicate that residue 206 is a highly occupied binding site for GFP in both the aqueous and lipid environments. Next, the strength of the interactions between monomers was examined. Decomposition of the binding free energy on a per-residue basis was determined using the molecular mechanics Poisson–Boltzmann surface area (MM-PBSA) method [18]. By combining the capabilities of MD simulation and single-molecule experiments, the data presented here elucidate important atomic features of the A206K mutation, which are key to preventing dimerization and give insights into the weak protein interactions on both the molecular and atomic levels.

## 2. Results

### 2.1. Single-Molecule Imaging of Wild-Type GFP and GFP Harboring the A206K Mutation (GFP-A206K)

**Figure 1:**
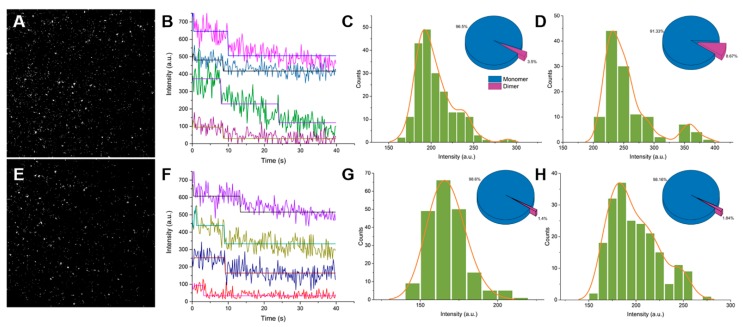
Single-molecule imaging of GFP and GFP-A206K. (**A**) Typical single-molecule fluorescence imaging of GFP on the coverslip surface. (**B**) One- and two-step photobleaching of GFP. Traces are arbitrarily shifted along the y-axis for display clarity. (**C**,**D**) Fluorescence intensity and photobleaching step distributions of GFP spots in 0.17 μg/mL and 0.57 μg/mL solutions, respectively. (**E**) Typical single-molecule fluorescence imaging of GFP-A206K on the coverslip surface. (**F**) One- and two-step photobleaching of GFP-A206K. Traces are arbitrarily shifted along the y-axis for display clarity. (**G**,**H**) Fluorescence-intensity and photobleaching step distributions of GFP-A206K spots in 0.17 μg/mL and 0.57 μg/mL solutions, respectively.

The dimerization of wild-type GFP in transgenic plant cells is due in part to residue A206 as discussed in our previous study [11]. To investigate the oligomerization of GFP, single-molecule fluorescence imaging was performed using an objective lens-type total internal reflection fluorescence microscope (TIRFM). As shown in Figure 1A,E, dispersed GFP and GFP-A206K with the diffraction-limited size were clearly identified. Most spots displayed a step-like photobleaching behavior, which confirmed that each spot contained only one GFP molecule both in the GFP and GFP-A206K solutions (Figure 1B). For better assessment of the GFP-oligomerization status, we extracted intensity time traces from each diffraction-limited fluorescent spot, revealing a stepwise photobleaching behavior (Figure 1B,E). At the lowest concentration (0.17 μg/mL), one-step bleaching showed that both GFP and GFP-A206K existed primarily as monomers (96.5 ± 3.3% and 98.6 ± 3.3%, respectively), with a low occurrence of two-step photobleaching spots indicative of dimers (3.5 ± 3.3% and 1.4 ± 3.3%, respectively) (Figure 1C,G). At higher concentrations (0.57 μg/mL) of GFP and A206K mutant, a shift in the distribution was only observed with wild-type GFP, with the percentage of two-step photobleaching spots dramatically increasing to 8.6 ± 2.7% (Figure 1D). Interestingly, the distribution did not shift in the GFP-A206K mutant, with two-step photobleaching spots observed only twice among a total of 982 spots analyzed (Figure 1E). Analysis of the fluorescence intensity distribution of GFP (Figure 1D) and GFP-A206K (Figure 1H) also showed different distributions in stoichiometry, and GFP had two distinct populations, including a minor population (4%) with a peak at 369 and an intensity approximately two-fold that of the major population (97%). For the GFP-A206K mutant, only one peak was observed corresponding to the monomeric form, indicating that no obvious oligomerization occurred between the mutant molecules at the concentrations evaluated (Figure 1G,H).

### 2.2. Interactions between GFP Molecules and Dimer Formation

**Figure 2:**
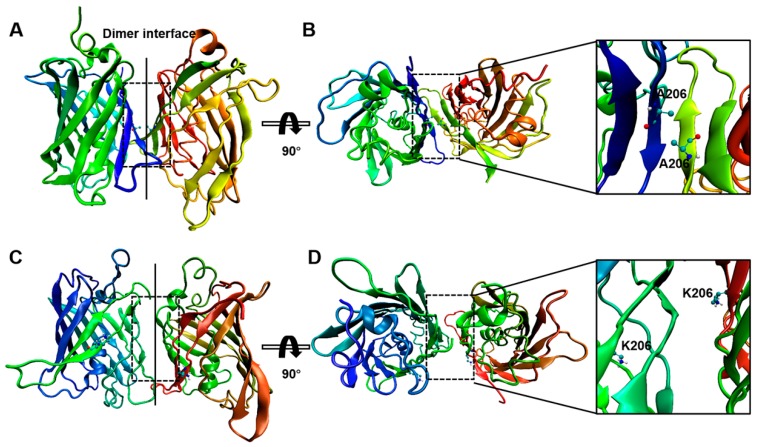
Analysis of the potential dimerization interfaces of wild-type GFP and GFP-A206K. (**A**) Representative structures of the most populated interfaces formed between wild-type GFP molecules in a water solution. (**B**) Top view of the GFP dimer by 90° rotation. (**C**) Representative structures of the most populated interfaces formed between GFP-A206K molecules in a water solution. (**D**) Top view of the GFP-A206K complex by 90° rotation.

Several studies have identified A206 as the key site for dimeric modulation of wild-type GFP [11]. To better understand the mechanism underlying this dimeric modulation of GFP, we performed 100-ns MD simulations examining systems composed of either wild-type or GFP-A206K monomers. A root mean squared deviation (RMSD) plot (Figure S1) reflected the time at which both models reached equilibrium: ~20 ns. Visual inspection of the equilibrated final structures of wild-type GFP revealed the amino acids that interact with their counterpart chain to provide a stable dimer (Figure 2A and Movie S1). Residue A206, present in the β10 strand, keeps the GFP atoms towards the interface exposed, enabling formation of hydrogen bonds (HBs) that significantly contribute to the head-to-head monomer interactions (Figure 2A,B). In contrast, simulations of the GFP-A206K mutant examining 20 trajectories over a 100-ns time scale did not detect any well-associated dimers, suggesting that the monomeric GFP-A206K exhibits much higher steric hindrance compared with wild-type GFP (Figure S2 and Movie S2). Each of these lysine residues brings a positive charge into close vicinity (1.9–2.6 Å) of each other in the hydrophobic patch forming the core of the dimer interface (Figure 2C,D). The distance between residues 206 and 207 in the core of the dimer interface is higher in GFP-A206K than in wild-type GFP in the MD trajectory (Figure S3). Such an extreme conformational change renders the formation of the dimer impossible at any concentration.

**Figure 3:**
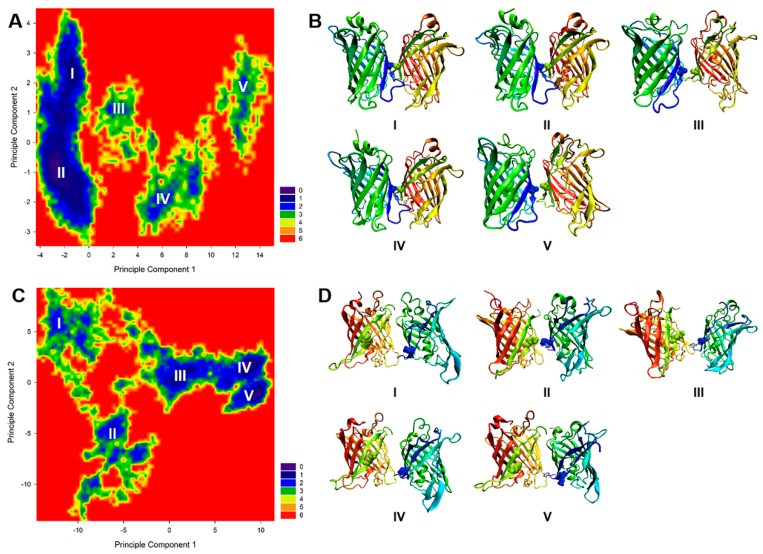
The folding population landscapes of GFP-206A and GFP-A206K projected onto the first two principle components. (**A**) Free energy landscape between the first and second principal components for GFP-206A. Regions of low free energy are indicated in cold colors (green to blue) and regions of high free energy in hot colors (orange to red). (**B**) Representative structures of the five highly-populated regions for GFP-206A. (**C**) Free energy landscape between the first and second principal components for GFP-A206K. (**D**) Representative structures of the five highly-populated regions for GFP-A206K.

To characterize the structural ensemble sampled during the MD simulation, we employed principal component analysis (PCA) to estimate the free energy landscape of the system at 293 K. The population matrix was then mapped onto a contour plot, as shown in Figure 3A,C. To find the representative structures of each local minimum, we performed clustering at each minimum using the GROMOS algorithm with the g_mmpbsa of GROMACS 2016.1. The binding energies estimated by MM-PBSA are shown in Table 1. Using this approach, five well-separated energy minima were identified. The representative structures of each GFP minimum are shown in Figure 3B. Structures I and II show that GFP can indeed form stable protein complexes with inter-chain β-sheets. Moreover, Structures III, IV, and V all contained a relatively high percentage of anti-parallel β-sheets; however, the global minimum of GFP-A206K Structures III, IV, and V consisted primarily of random coils and turns, with the exception of a short segment of β-sheets formed from residue 206K (Figure 3D). The estimated binding energy using MM-PBSA showed a relatively large contribution from van der Waals (vdW) interactions, which confirmed that hydrophobic effects are the main driving force (Table 1).

### 2.3. N-Myristoylation Targets GFP to the Plasma Membrane

**Figure 4:**
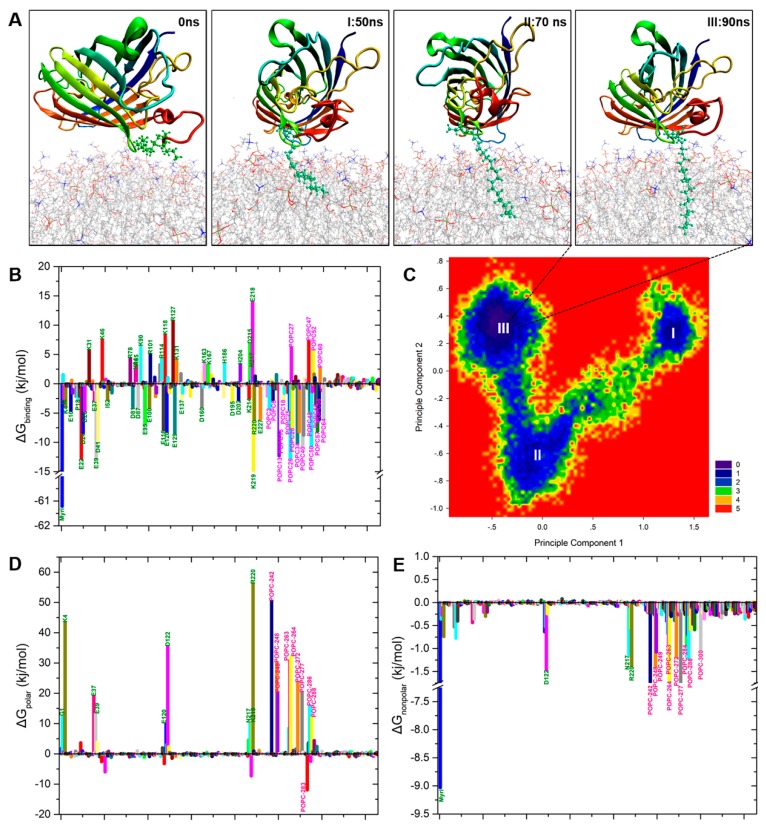
Atomic MD simulations reveal how the myristoyl group anchors GFP to a POPC membrane. (**A**) Snapshots showing the process of myristoyl group insertion into the lipid bilayer. The myristoyl moiety is highlighted in green and displayed in Corey-Pauling-Koltun (CPK) mode. (**B**) Binding free energy contributions of per-residue (olive green) and each POPC lipid (purple) to the formation of the protein-lipid bilayer complex. Error bars were not included since the standard errors were always considered in GROMACS algorithms. (**C**) Contour plot of the relative free energy landscape (FEL) determined from principal component analysis (PCA) using the first and second principal components for the myristoylated GFP-POPC system. The energy scale is given in kJ/mol, with red indicating the high free energy regions and blue the low free energy regions, corresponding to unfavorable and favorable conformations, respectively. Representative structures of the three highly-populated regions, I (50 ns), II (70 ns), and III (90 ns), with the energy minimum are also shown in (**A**). (**D**) Polar binding energy contribution of each residue in the myri-GFP-POPC system. (**E**) Nonpolar binding energy contribution of each residue in the myri-GFP-POPC system. The residues with the most favorable (<−10 kJ/mol) contributions and unfavorable (>10 kJ/mol) are labeled.

Our atomic simulations captured multiple instances of spontaneous membrane insertion of the myristoyl moiety. In all of these cases, after initial reorientation, the N-terminal domain of myristoylated GFP was presented to the lipid bilayer prior to myristoyl insertion. Once the methyl end of the myristoyl chain approached a sufficient gap between lipid head groups, it penetrated the carbonyl region of the bilayer and became embedded among the acyl chains of the surrounding phospholipids (Figure 4A and Movie S3). Notably, we observed membrane insertion of the myristoyl moiety for several, but not all, of the trajectories. In several cases, myristoylated GFP approached the lipid bilayer with an unfavorable orientation, interacting with lipid head groups via its positively-charged residues, leading to the detachment of myristoylated GFP from the lipid bilayer.

To assess the importance of the myristoyl anchor for membrane binding of GFP, we deleted the myristoyl group from the final snapshot of a trajectory featuring myristoyl insertion and continued the simulation. We found that without the myristoyl anchor, the orientation of GFP relative to the membrane was destabilized, ultimately resulting in GFP detaching itself from the membrane surface (see Figure S4 and Movie S4). To further quantify the free energy gain resulting from myristoyl insertion, we employed MM-PBSA to calculate the free energy profile of the system (Figure 4B,D,E). As an alternative determination of the free energy landscape, we performed principal component analysis of the configurational ensembles in the lowest binding free energy. The first two principal components provided an optimal separation of conformations and effectively described the entire free energy ensemble in three general configurations, in which the myristoyl moiety was able to insert itself into the POPC (2-oleoyl-1-palmitoyl-sn-glycero-3-phosphocholine) lipid bilayer (Figure 4C). Taken together, these results underscore the crucial importance of the myristoyl moiety for GFP association with the membrane.

### 2.4. Binding Free Energy Analysis of Dimerization

**Figure 5:**
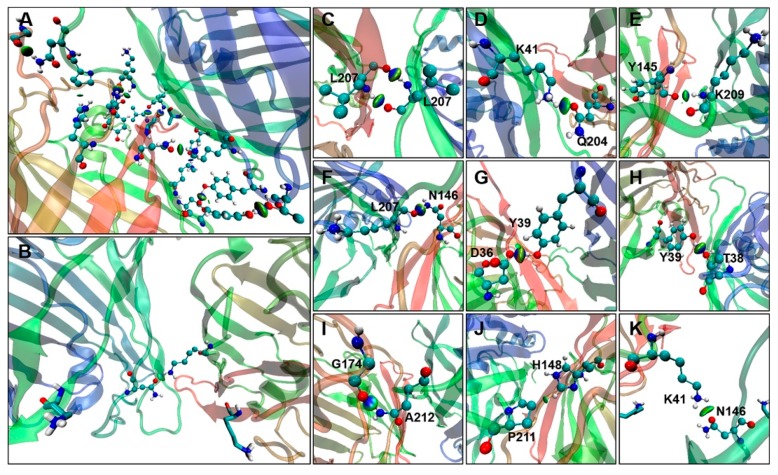
The 3D isocontours generated using an independent gradient model (IGM) represent the non-covalent interactions (NCIs) formed between GFP molecules. Green areas correspond to λ2 ≈ 0 (weak). λ2 is one of the three eigenvalues of the electron-density Hessian matrix, with λ1 ≤ λ2 ≤ λ3. All isosurfaces are colored according to a blue-green-red scheme over a range of −0.1 < *sign*(λ2)ρ < 0.1 a.u. (**A**) The interface region and isosurface map between two GFP monomers. (**B**) The interface region and isosurface map between two GFP-A206K monomers. (**C**–**J**) The key non-covalent interaction and isosurface map of residues near A206 at the GFP-dimer interface. (**K**) The non-covalent interaction and isosurface map between K41 and N146 of GFP-A206K.

To better understand the modulatory role of residue 206 in GFP dimerization, weak interactions were analyzed in the GFP dimer models by means of non-covalent interaction (NCI) analysis. NCI plots show a cascade of non-covalent interactions between the dimer interface and residues 207, 204, 38, 41, 36, and 39. For example, around the L207–L207 (dimer interface), residues 204–41, and residues 209–148’s interactions were mediated by the formation of HBs, resulting in additional interactions between GFP molecules (Figure 5C–J).

As shown in Figure 5A, there is a key non-covalent interaction between the hydrophobic side-chain of residue 207 (which is a point of contact within the dimer interface) and a side-chain of residue 204 (Figure 5C,D,F). Furthermore, the point of contact between the β10-strand and β11-strand, K209, is shown to interact strongly via its side-chain C=O group with the –NH_3_ group of Tyr145 and to form a strong HB via its –NH_2_ group with the backbone C=O of Asn146. Furthermore, Gln204 in the β10-strand also forms a strong HB with Lys41 via its –NH2 group (NH…O: 2.1 Å). Despite the variety of residues playing a role in the activation process, the optimized A206K model shows a monomeric status on different trajectories, suggesting that the geometrical arrangement of these HB and vdW interactions is a key feature that differentiates active interactions.

Monitoring the interactions between the residues of GFP-A206K using independent gradient model (IGM) analysis provided evidence of weak interactions during dimer formation (Figure 5B). Using this approach, at the very end of the MD simulation, the two Lys206 residues adopted a particular orientation in the chosen MD trajectories, positioning their side chains away from each other. This particular change in positioning also led to hydrophobic interactions with Asn146 and Lys41 (Figure 5K). As shown in Figure 5B, only one HB was found in GFP-A206K at the end of the MD simulation. The nitrogen and hydrogen atoms of Lys41 form dynamic HBs with the hydrogen atom of Asn146 (Figure 5B,K). From these analyses, it is obvious that the β10-strand is the primary mediator of the interactions between particular residues in GFP monomers.

### 2.5. Binding Free Energy Analysis of Membrane Insertion

To provide further insight into the interactions between GFP monomers, as well as between myristoylated GFP and lipid membranes, the binding free energies of GFP dimers and the myristoylated-GFP–membrane complex were calculated using the MM-PBSA method. Using the most stabilized orientation of the proteins and membrane in our simulation, we calculated the binding free energy of GFP dimers and the myristoylated-GFP–membrane complex to identify the detailed features of the interactions. Table 1 shows the free energy components of GFP dimers and the myristoylated-GFP–membrane complex obtained from the MM-PBSA calculations. The results show that both ΔG_polar_ and ΔG_nonpolar_ favor the dimerization of GFP and binding of myristoylated GFP to the lipid membrane; however, ΔG_polar_ (736.2 kJ/mol for dimer and 341.5 kJ/mol for membrane) was much stronger than ΔG_nonpolar_ (−251.3 kJ/mol for dimer and −57.8 kJ/mol for membrane), suggesting that the nonpolar interactions are a key factor in the formation of both interactions.

**Figure 6:**
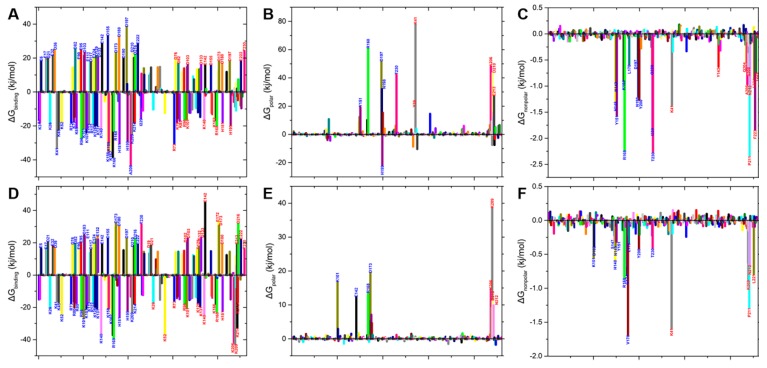
Per-residue energy contribution plots. Binding free energy contribution of each residue to the interactions between GFP monomers (**A**) and GFP-A206K monomers (**D**). Polar binding energy contribution of each residue to the interactions between GFP monomers (**B**) and GFP-A206K monomers (**E**). Nonpolar binding energy contribution of each residue to the interactions between GFP monomers (**C**) and GFP-A206K monomers (**F**). The residues with the most favorable (<−10 kJ/mol) and unfavorable (> 10 kJ/mol) contributions are labeled.

We next performed a free energy decomposition analysis of each residue in GFP and myristoylated GFP to identify the residues critical for the interactions between GFP monomers and between myristoylated GFP and the membrane (Figure 6A,D and Figure 4B). In the free energy decomposition analysis, the important residues were defined as those with an absolute value of free energy contribution of >10 kJ/mol, a commonly-used threshold for decomposition analysis. To describe the free energy decomposition in detail, the energy contributions of the polar and the nonpolar energies were analyzed separately.

First, we analyzed the per-residue polar energy contributions. Effective polar interaction contributions were obtained from the majority of charged residues in both interactions (Figure 6A,D and Figure 4B). The energy contributions of both positively- and negatively-charged residues, such as R168, K41, E213, and D210, were found to be unfavorable, whereas those of positively-charged residues, such as K79, R80, and H199, were favorable (Figure 6A) in the interaction between GFP-A206K molecules (Figure 6D) and in membrane binding (Figure 4B).

Finally, the nonpolar energy contributions are presented in Figure 6C,F, and Figure 4E. As the data presented here suggest that polar interactions are not the driving force behind dimer formation in any of the models tested, we next evaluated the contribution of nonpolar interactions related to hydrophobicity in selectivity. In the GFP dimerization simulation (Figure 6C), the most significant nonpolar interactions were associated with residues Tyr151, Arg168, Pro211, Tyr200, Phe223, and Thr230. In the myristoylated-GFP–membrane simulation (Figure 4E), residues Asp122, Asn217, and Arg220 all had favorable nonpolar interactions. Finally, residue Lys141 provided both polar and nonpolar interactions, indicating that these residues are possibly involved in GFP dimerization. On the other hand, the contributions of Lys41, Arg168, and Thr230 to the interactions between GFP monomers were distinctly different from those between GFP-A206K monomers. As shown in Figure 4B,E, both the polar and nonpolar contributions of protein residues were markedly decreased in the myristoylated-GF–membrane simulation compared with the GFP dimerization simulation. These findings suggest that the electrostatic interactions between residues are a key factor involved in GFP dimerization.

## 3. Discussion

The ability to detect and characterize the complex behavior of living cells at the single-molecule level has made great progress in recent years due to advancements in genetically-encoded FPs and new imaging techniques. Single-molecule imaging and tracking have enabled examination of the structure, dynamics, and interactions of molecules in their native environments, revealing information that would have been lost in bulk ensemble averages [19,20,21]. Most of the native FPs isolated to date exist naturally as oligomeric complexes [2,3]; therefore, selecting monomeric FPs is critical when designing FP-fusion proteins and single-molecule reporters. Inappropriate interactions [8], organelle reorganization [7], and subunit stoichiometry [22] are some of the adverse consequences of using non-monomeric FPs. To maximize FP utility and avoid undesirable artifacts, it is critical to have a more accurate picture of the underlying mechanism responsible for GFP dimerization and membrane binding under physiological conditions. In this work, we utilized a combination of mutagenesis and single-molecule studies in combination with atomistic MD simulations to elucidate the mechanisms underlying the formation of protein dimers and protein–membrane complexes. Surprisingly, we found that the high-affinity GFP dimer-binding site is located in the β-sheet of GFP and that the protein–membrane contact sites only bind phosphoinositide-rich membranes with low affinity. We also identified specific binding sites and characterized the binding free energy landscape of GFP dimers and the myristoylated-GFP–membrane complex. These data will enable a better understanding of the protein oligomerization process and macromolecular weak interactions, which will improve FP design and development.

In our previous study, we observed that the A206K mutation alone dramatically reduced the rate of GFP dimerization in living plant cells [11]. The propensity of a particular molecule to form a dimer depends on its molecular affinity, termed the dissociation constant or K_d_, and its concentration. We calculated the observed dimerization probability within a certain time at different concentrations of GFP and GFP-A206K using TIRFM (Figure 1). A key step in achieving single-fluorophore detection in a solution is to decrease the background fluorescence in the bulk solution. TIRFM is among the most popular options for single-molecule imaging [19,23,24] and was the method of choice for these analyses. Using single-molecule imaging in combination with photobleaching, we found that only one photobleaching step was necessary for the GFP-A206K mutant, confirming that GFP-A206K remains monomeric at high concentrations.

Elucidation of the underlying mechanism of dimerization requires a detailed understanding of how conformational flexibility affects interactions and structural changes at a resolution not readily achieved with most experimental methods. MD simulation is a powerful tool for understanding the dynamic interactions of proteins with their molecular partners and environment [25]. This approach enabled us to probe the conformational dynamics and electrostatic interactions associated with various aspects of protein function, although it is less effective for processes that occur over longer time scales, such as transporter turnover [26,27]. Our MD results indicated that a pair of wild-type GFP molecules exhibits stable two-stranded parallel or antiparallel β-sheets, across two chains of the dimer interface. These constructs were present 80% of the time during the last 100 ns of the simulations. In contrast, for the A206K mutant, each of the substituted lysine residues brings a positive charge into close vicinity (1.9–2.6 Å) of the hydrophobic patch forming the dimer interface core. As such mutations have no effect other than on the dimerization rate of GFP, we recommend including them (preferably A206K) in all GFP expression constructs where dimerization is not desirable.

*N*-myristoylation is a co-translational lipid anchor modification of eukaryotic and viral proteins that plays a vital role in cellular signaling, protein–protein interactions, and targeting of proteins to the endomembrane and plasma membrane [28]. The C14:0 saturated fatty acid myristate is linked via an amide bond to the N-terminal glycine residue of the target protein, which is often part of the MGXXX(S/T) consensus sequence [29,30]. *N*-myristoylation is required, but not sufficient for membrane anchoring and often occurs together with S-acylation of proximal cysteine residues [31]. In our previous experimental study, we observed that a modified GFP harboring a myristoyl binding site (GIGKSK) was efficiently localized to the plasma membrane in plant cells [11]. To gain atomistic insight into the membrane binding of *N*-myristoylated GFP, we performed atomic MD simulations of a myristoylated GFP molecule in the proximity of a POPC (1-palmitoyl-2-oleoyl-*sn*-glycero-3-phosphocholine) bilayer. These simulations demonstrated that myristoylation of the glycine residue of GFP efficiently increases its affinity to the plasma membrane. A dynamic interaction between the myristoyl moiety and polar headgroups of the phospholipids not evident in the frozen crystal structure was also observed. Importantly, our additional quantification of the free energy for myristoylated GFP insertion into the lipid bilayer showed a free energy preference for the membrane over the aqueous phase (Table 1), which indicates spontaneous anchoring and insertion of the construct mediated by the myristoyl moiety. These findings allow us to establish the key factors governing successful membrane insertion of the myristoyl moiety and parse the relative contributions of hydrophobic and electrostatic interactions to the reversible membrane binding of the protein.

As the mechanisms by which different residues interact during GFP dimerization are still largely unknown, it is important to look closer at the origins of the weak interactions involved in dimerization. NCIs are of paramount importance in weak interaction analysis, playing a key role in the interaction and recognition of biomolecules, as well as in the formation of biomolecular structures and clusters [32]. This class of interactions spans a wide variety of attraction (vdW and HB) and repulsion (steric) forces between atoms or molecules based on electron density properties [33]. However, the initial NCI approach does not provide an absolute means of distinguishing attractive and repulsive contributions in intramolecular and intermolecular interactions, which is of crucial importance in the analysis of big systems. A new IGM developed by Corentin et al. [34] allows for quantitative comparisons of the strength of NCIs by calculating the IGM descriptor δg, which corresponds directly to the charge density gradient(s) in real space. Applying IGM analysis (Figure 5), numerous vdW and HB interactions involving residues Gln204, Leu207, and Lys209 at the dimer interface were identified and corroborated (Figure 2). Remarkably, the repulsive interactions can maintain separation between GFP-A206K monomers at the dimer interface, even though the vdW and HB interactions still exist among residues Lys41 and Asn146 (Figure 5).

The structural and energetic requisites underlying biomolecular interactions, such as those involved in recognition or catalysis, can be quantified in terms of their binding free energies, with a variety of computational approaches available to calculate these estimates. Although there are more rigorous methods of calculating binding energies, such as free energy perturbation (FEP) and thermodynamic integration (TI), they are of limited use for larger systems due to their expensive computational costs [35,36]. To investigate the binding affinities between GFP monomers and between myristoylated GFP and the membrane, the MM-PBSA method was used to calculate binding energy using the g_mmpbsa code distributed by Kumari [18]. This method can also be used to estimate the energy contribution per residue to the binding energy. The binding energy calculations showed that residues R168, P211, and T230 in wild-type GFP and residues K101, R168, and D17316 in the A206K mutant play important roles in the formation of the protein complex (Figure 6).

In this study, our computed binding energies for GFP dimers were highly consistent with experimentally-derived values [2]. These results clearly demonstrate why the A206K mutation, which causes intense electrostatic repulsion at the dimer interface (Figure 6), dramatically impairs dimer formation between GFP-A206K molecules. It is notable that, in several cases, myristoylated GFP fails to insert into the lipid bilayer even though the binding free energy of the final simulation complex is relatively low (Figure 6, Table 1). An unfavorable orientation or large distance between myristoylated GFP and the membrane can diminish the likelihood of membrane insertions. Alternatively, reasonable positioning may be necessarily optimized and localized at the beginning of the MD simulations. This indicates that specific interactions of macromolecules within the cell define the most favorable orientation among them.

## 4. Materials and Methods

### 4.1. Recombinant GFP Expression and Purification

To express and purify proteins, the ORFs of wild-type GFP and GFP-A206K were subcloned into pET28b(+) and transformed into *E. coli* strain BL21DE3(+). Bacteria were then pelleted by centrifugation, resuspended in 20 mM Tris–HCl pH 7.4, and lysed by sonication. Bacterial lysates were cleared by centrifugation at 12,000× *g* for 10 min at 4 °C. The 6× His-tagged proteins were affinity-purified on Ni-NTA-agarose (Qiagen China, Shanghai, China) columns. Both total cell and purified proteins were analyzed by sodium dodecyl sulfate, polyacrylamide gel electrophoresis (SDS-PAGE, 12% resolving; 5% stacking).

### 4.2. Single Molecule Imaging and Analysis

The total internal reflection fluorescence microscope (TIRFM) was used to visualize single GFP molecules based on an inverted microscope (IX-71; Olympus, Tokyo, Japan), equipped with a laser-based TIRFM illumination module (IX2-RFAEVA-2; Olympus) and an oil objective (numerical aperture of 1.45, Olympus). GFP was excited by a 473-nm laser with average laser intensity setting to 0.2 μW/μm^2^, and high-quality dichroic mirrors and filters were used to maximize the yield of photons, which were detected with a back-illuminated electron-multiplying charge-coupled device (EMCCD) camera (ANDOR iXon DV897D-CS-BV; Andor Technology, Belfast, UK). Movies of 200–300 frames were acquired using a frame rate of 10–20 Hz. Single particle tracking (SPT) and photo-bleaching steps analysis were performed via custom-built MATLAB scripts.

### 4.3. Atomistic Molecular Dynamics Simulation

GROMACS 2016.1 was used for all atomistic molecular dynamics simulations, with the GROMOS96 united atom force field and the SPC water model [37]. The simulations were performed at a temperature of 300 K using a Berendsen thermostat with τp = 0.1 ps. A constant pressure of 1 bar was maintained with an isotropic coupling constant τP) 1.0 ps and compressibility = 4.5 × 10^−5^ bar^−1^. The integration time step was 2 fs. The LINCS method was used to constrain bond lengths. Coordinates were saved every 2 ps for analysis.

### 4.4. Calculation of Absolute Binding Free Energies

The binding free energy was calculated based on the molecular mechanics Poisson−Boltzmann surface area (MM-PBSA) method [38,39] with the g_mmpbsa code [18]. The detailed calculation was based on previous reports in the literature [40,41]. The long-range forces, such as an der Waals interactions, were identified with Multiwfn [42], and the independent gradient model, as well as isosurface defining the weak interactions was plotted using Multiwfn and VMD [43] software.

### 4.5. Principal Component Analysis

Principal component analysis (PCA) was performed to predict the collective motion of atoms and simplify large, complex MD datasets within the GROMACS 2016.1 package [37]. A covariance matrix was constructed over the equilibrated simulation time through diagonalization of the symmetric matrix, from which a set of eigenvectors and eigenvalues was identified. The essential subspace relevant to the GFP, GFP-206K, and myri-GFP-POPC systems was constructed by projecting the PCA along the most important eigenvectors from the analysis using the g_covar and g_anaeig GROMACS 2016.1 package utilities.

## 5. Conclusions

Single-molecule imaging, MD simulation, weak interactions, and free energy calculations were performed to enhance characterization of the interactions between GFP monomers and to explore the molecular mechanism underlying insertion of *N*-myristoylated GFP into the plasma membrane. We elucidated the molecular and atomistic details of these processes under varying conditions. Direct single-molecule imaging and tracking demonstrated that wild-type GFP has a tendency to form dimers, whereas the A206K mutation dramatically reduced GFP dimerization. The results from MD simulations and weak interaction analysis indicated that vdW and HB interactions play an important role in GFP dimerization. IGM analysis also indicated that GFP residues Gln204, Leu207, and Lys209 form stronger vdW and HB interactions in wild-type GPF than in GFP-A206K. The free energy calculation showed that Glu and Asp are important residues for *N*-myristoylated GFP insertion into the membrane. Our MD simulations not only relate directly to the single-molecule experiments described here, but also provide a detailed interpretation of the dimerization and membrane insertion processes in terms of the underlying mechanisms, which is not possible using standard experimental methods.

In summary, this study provides a combination of single-molecule observations and multiple MD simulations allowing elucidation of the mechanism underlying protein dimerization and membrane insertion, providing a greater understanding of the key factors regulating their biological function.

## Figures and Tables

**Table 1:** Binding free energy (kJ/mol) for the dimerization of GFP, GFP-A206K monomer, and insertion of myristoylated GFP in the lipid bilayer and contributions of solvation, van der Waals, and electrostatic interactions, and entropic terms using the same trajectory method. POPC, 2-oleoyl-1-palmitoyl-sn-glycero-3-phosphocholine.

	GFP-A206	GFP-K206	Myristoylated-GFP–POPC
ΔE_elec_	−252.8 ± 25.6	−73.4 ± 72.5	−286.4 ± 28.9
ΔE_vdW_	−344.6 ± 17.5	−89.3 ± 12.4	−238.9 ± 35.3
ΔG_polar_	736.2 ± 74.8	217.9 ± 103.2	341.5 ± 126.8
ΔG_nonpolar_	−251.3 ± 49.4	−18.1 ± 2.6	−57.8 ± 6.8
ΔG_binding_	−112.5 ± 43.7	37.1 ± 15.4	−241.6 ± 90.9

Energy values are rounded to one decimal place and shown in kilojoules per mol. The binding energy was divided into individual components using g_mmpbsa, where ΔE_elec_ is the molecular mechanics (MM) electrostatic energy, ΔE_vdW_ is the MM van der Waals energy, ΔG_polar_ is the polar solvation energy, and ΔG_nonpolar_ is the nonpolar solvation energy.

## Supplementary Materials

Supplementary materials can be found at https://www.mdpi.com/1422-0067/20/6/1410/s1. Figure S1. (A) RMSD plot of GFP and GFP-A206K and (B) myristoylated GFP with the POPC lipid bilayer. The values of RMSD demonstrate that after 20 ns of MD simulation, the GFP and GFP-A206K structure reaches a balance of stability. However, the myristoylated GFP and POPC system reaches the balance of stability after 70 ns. Figure S2. The steric clashes between two lysine chains (206K) makes an unfavorable contact in the interface of GFP-A206K molecules. Figure S3. Distance analysis between residues 206-206, 207-207 in GFP (A and B) and GFP-A206K (C and D), respectively. Figure S4. Snapshots showing the detachment of GFP from the POPC lipid bilayer after deleting the myristoyl group. Movie S1. Molecular dynamics simulations showing the dimerization of wild-type GFP. Movie S2. Molecular dynamics simulations showing that the A206K single mutation inhibited GFP-A206K to form stable dimer. Movie S3. All-atom molecular dynamics simulations of the insertion of *N*-myristoylated GFP to a lipid bilayer. Movie S4. All-atom molecular dynamics simulations showing that without the myristoyl anchor, the orientation of GFP relative to the lipid bilayer became destabilized, and finally detached completely from the membrane surface.
