# Supplementary figures and images for: Single-Molecule Imaging and Computational Microscopy Approaches Clarify the Mechanism of the Dimerization and Membrane Interactions of Green Fluorescent Protein
Source: Int J Mol Sci. 2019 Mar 20;20(6):1410. doi: 10.3390/ijms20061410 (PMC6471090; doi:10.3390/ijms20061410)

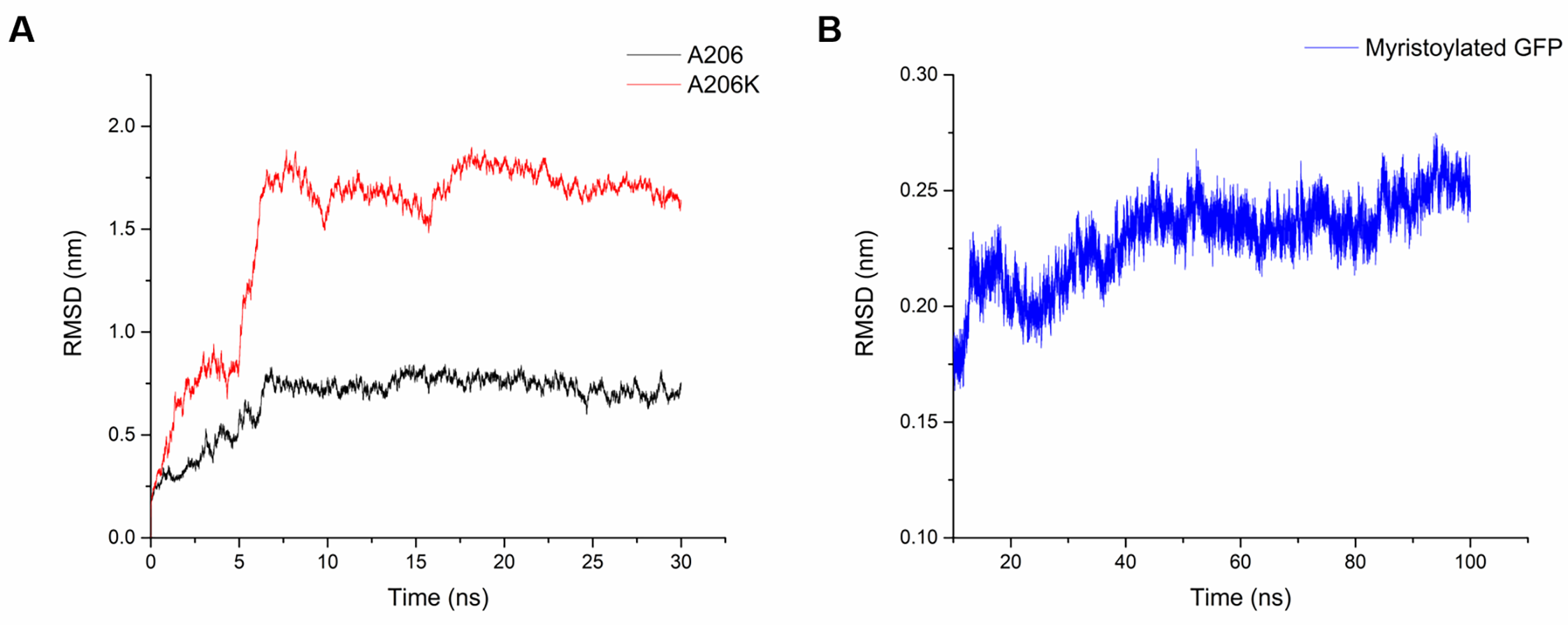

Supplement: Supplementary file 1 [file ijms-20-01410-s001.zip › ijms-467303-SI/Supplementary Files/Figure S1.tif]

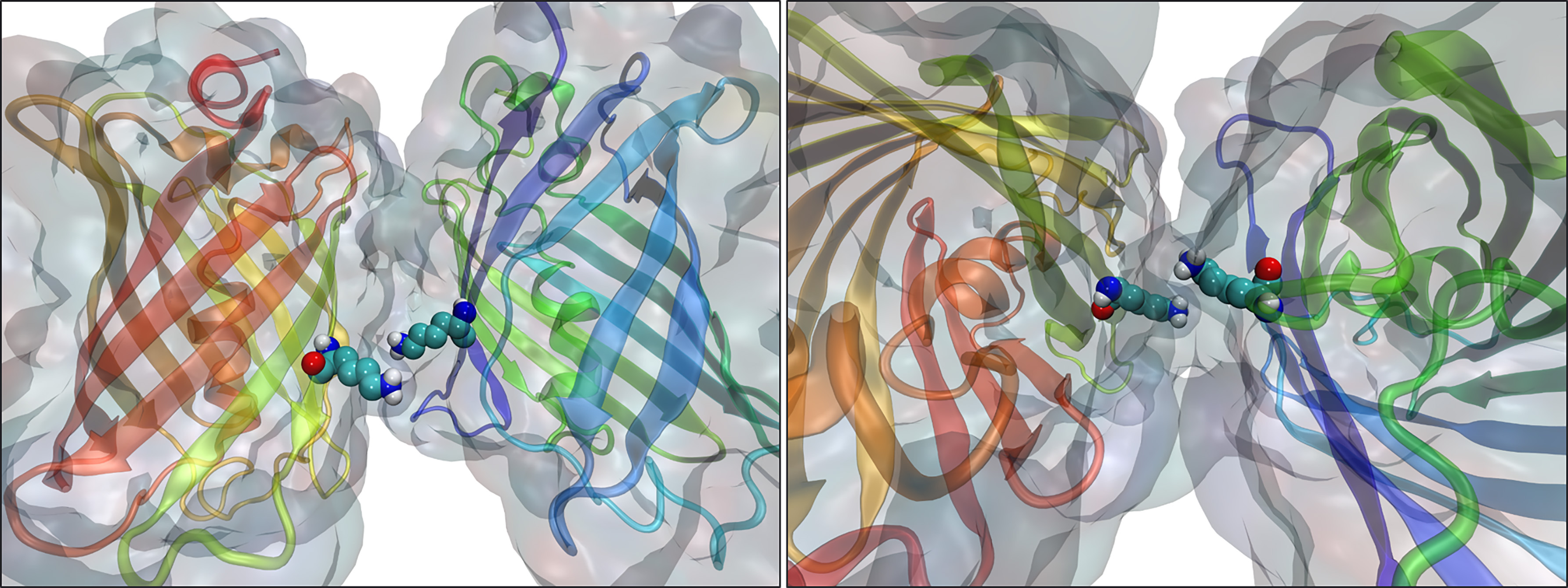

Supplement: Supplementary file 1 [file ijms-20-01410-s001.zip › ijms-467303-SI/Supplementary Files/Figure S2.jpg]

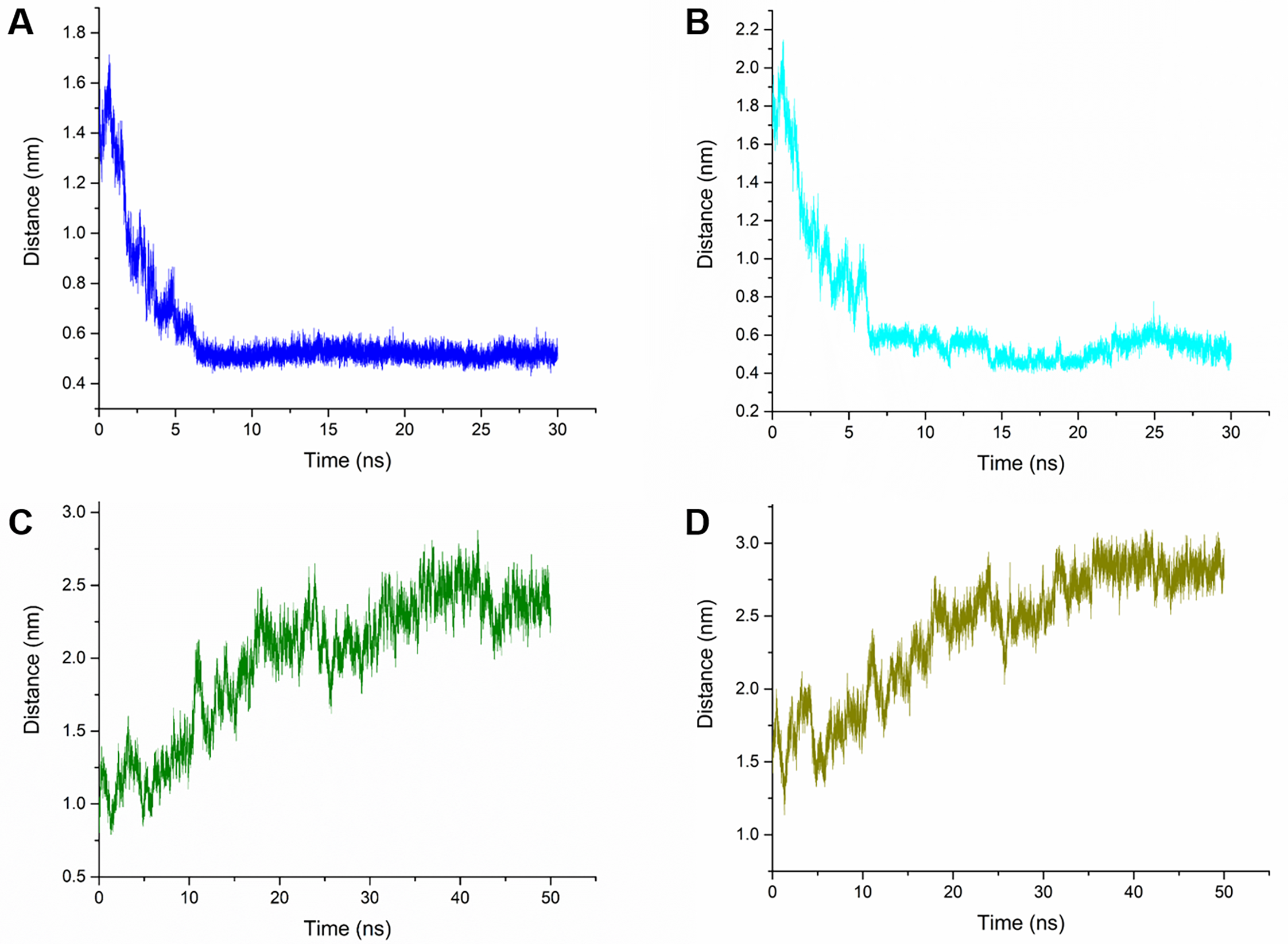

Supplement: Supplementary file 1 [file ijms-20-01410-s001.zip › ijms-467303-SI/Supplementary Files/Figure S3.tif]

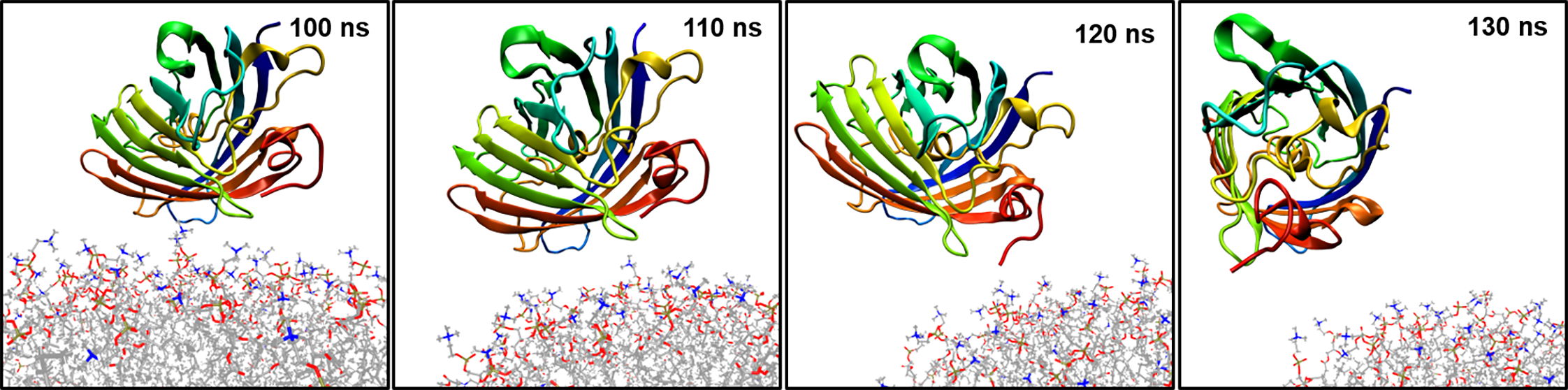

Supplement: Supplementary file 1 [file ijms-20-01410-s001.zip › ijms-467303-SI/Supplementary Files/Figure S4.tif]
